# Supplementary material for: Multi‐dataset identification of innovative feature genes and molecular mechanisms in keratoconus
Source: J Cell Mol Med. 2024 Sep 19;28(18):e70079. doi: 10.1111/jcmm.70079 (PMC11412914; doi:10.1111/jcmm.70079)
Supplement: Supplementary file 2 — Material S2. [file JCMM-28-e70079-s002.zip › 7.GSEA analysis/2.ATOH7/KEGG_TRYPTOPHAN_METABOLISM.html]

Details for gene set KEGG\_TRYPTOPHAN\_METABOLISM[GSEA]

|  || Dataset | ATOH7.group.cls#h\_versus\_l |
| Phenotype | group.cls#h\_versus\_l |
| Upregulated in class | h |
| GeneSet | KEGG\_TRYPTOPHAN\_METABOLISM |
| Enrichment Score (ES) | 0.42200252 |
| Normalized Enrichment Score (NES) | 1.3819385 |
| Nominal p-value | 0.054766733 |
| FDR q-value | 0.331172 |
| FWER p-Value | 0.918 |
Table: GSEA Results Summary

  

Fig 1: Enrichment plot: KEGG\_TRYPTOPHAN\_METABOLISM      
 Profile of the Running ES Score & Positions of GeneSet Members on the Rank Ordered List

  

| SYMBOL | TITLE | RANK IN GENE LIST | RANK METRIC SCORE | RUNNING ES | CORE ENRICHMENT || 1 | ALDH2 | na | 221 | 0.659 | 0.1169 | Yes |
| 2 | KYNU | na | 305 | 0.623 | 0.2311 | Yes |
| 3 | CYP1B1 | na | 1436 | 0.393 | 0.2719 | Yes |
| 4 | CYP1A1 | na | 1988 | 0.345 | 0.3206 | Yes |
| 5 | AOX1 | na | 2532 | 0.307 | 0.3623 | Yes |
| 6 | AADAT | na | 3628 | 0.250 | 0.3775 | Yes |
| 7 | IDO1 | na | 3694 | 0.248 | 0.4220 | Yes |
| 8 | ASMT | na | 5755 | 0.178 | 0.3959 | No |
| 9 | ALDH9A1 | na | 7741 | 0.125 | 0.3620 | No |
| 10 | ALDH1B1 | na | 7850 | 0.122 | 0.3816 | No |
| 11 | KMO | na | 8225 | 0.114 | 0.3921 | No |
| 12 | EHHADH | na | 8475 | 0.109 | 0.4053 | No |
| 13 | TPH1 | na | 8824 | 0.102 | 0.4143 | No |
| 14 | GCDH | na | 9361 | 0.093 | 0.4162 | No |
| 15 | OGDH | na | 11020 | 0.066 | 0.3807 | No |
| 16 | IL4I1 | na | 11846 | 0.055 | 0.3671 | No |
| 17 | CAT | na | 13546 | 0.035 | 0.3247 | No |
| 18 | HADH | na | 13799 | 0.033 | 0.3235 | No |
| 19 | ALDH7A1 | na | 14436 | 0.026 | 0.3101 | No |
| 20 | ACAT1 | na | 15830 | 0.014 | 0.2726 | No |
| 21 | INMT | na | 16192 | 0.011 | 0.2643 | No |
| 22 | HADHA | na | 16925 | 0.005 | 0.2442 | No |
| 23 | ECHS1 | na | 16985 | 0.005 | 0.2433 | No |
| 24 | WARS2 | na | 17612 | -0.001 | 0.2254 | No |
| 25 | TDO2 | na | 18508 | -0.009 | 0.2012 | No |
| 26 | AFMID | na | 18650 | -0.010 | 0.1990 | No |
| 27 | MAOA | na | 19096 | -0.014 | 0.1887 | No |
| 28 | HAAO | na | 19260 | -0.015 | 0.1869 | No |
| 29 | ALDH3A2 | na | 20819 | -0.030 | 0.1475 | No |
| 30 | CYP1A2 | na | 21705 | -0.039 | 0.1294 | No |
| 31 | AOC1 | na | 22219 | -0.045 | 0.1231 | No |
| 32 | ACMSD | na | 23236 | -0.058 | 0.1046 | No |
| 33 | ACAT2 | na | 23817 | -0.067 | 0.1004 | No |
| 34 | DDC | na | 25136 | -0.088 | 0.0788 | No |
| 35 | OGDHL | na | 25257 | -0.090 | 0.0922 | No |
| 36 | AANAT | na | 27341 | -0.134 | 0.0572 | No |
| 37 | IDO2 | na | 28666 | -0.169 | 0.0506 | No |
| 38 | MAOB | na | 32336 | -0.315 | 0.0038 | No |
| 39 | TPH2 | na | 32777 | -0.348 | 0.0562 | No |
Table: GSEA details [plain text format]

  

Fig 2: KEGG\_TRYPTOPHAN\_METABOLISM      
 Blue-Pink O' Gram in the Space of the Analyzed GeneSet

  

Fig 3: KEGG\_TRYPTOPHAN\_METABOLISM: Random ES distribution      
 Gene set null distribution of ES for **KEGG\_TRYPTOPHAN\_METABOLISM**
